# Correlated cryo-SEM and CryoNanoSIMS imaging of biological tissue

**DOI:** 10.1186/s12915-023-01623-0

**Published:** 2023-06-07

**Authors:** Anders Meibom, Florent Plane, Tian Cheng, Gilles Grandjean, Olivier Haldimann, Stephane Escrig, Louise Jensen, Jean Daraspe, Antonio Mucciolo, Damien De Bellis, Nils Rädecker, Cristina Martin-Olmos, Christel Genoud, Arnaud Comment

**Affiliations:** 1 Laboratory for Biological Geochemistry, School of Architecture, Civil and Environmental Engineering, École Polytechnique Fédérale de Lausanne (EPFL), Lausanne, CH-1015 Switzerland; 2 Center for Advanced Surface Analysis, Institute of Earth Sciences, University of Lausanne, Lausanne, CH-1015 Switzerland; 3 Mechanical Workshop, School of Basic Sciences, École Polytechnique Fédérale de Lausanne, Lausanne, CH-1015 Switzerland; 4 Electron Microscopy Facility, University of Lausanne, Lausanne, CH-1015 Switzerland; 5 Institute of Physics, School of Basic Sciences, École Polytechnique Fédérale de Lausanne, Lausanne, CH-1015 Switzerland

**Keywords:** High pressure freezing, *Hydra viridissima*, Isotope labeling, NanoSIMS, Osmoregulation, Photosymbiosis

## Abstract

**Background:**

The development of nanoscale secondary ion mass spectrometry (NanoSIMS) has revolutionized the study of biological tissues by enabling, e.g., the visualization and quantification of metabolic processes at subcellular length scales. However, the associated sample preparation methods all result in some degree of tissue morphology distortion and loss of soluble compounds. To overcome these limitations an entirely cryogenic sample preparation and imaging workflow is required.

**Results:**

Here, we report the development of a CryoNanoSIMS instrument that can perform isotope imaging of both positive and negative secondary ions from flat block-face surfaces of vitrified biological tissues with a mass- and image resolution comparable to that of a conventional NanoSIMS. This capability is illustrated with nitrogen isotope as well as trace element mapping of freshwater hydrozoan Green Hydra tissue following uptake of ^15^N-enriched ammonium.

**Conclusion:**

With a cryo-workflow that includes vitrification by high pressure freezing, cryo-planing of the sample surface, and cryo-SEM imaging, the CryoNanoSIMS enables correlative ultrastructure and isotopic or elemental imaging of biological tissues in their most pristine post-mortem state. This opens new horizons in the study of fundamental processes at the tissue- and (sub)cellular level.

**Teaser:**

CryoNanoSIMS: subcellular mapping of chemical and isotopic compositions of biological tissues in their most pristine post-mortem state.

**Supplementary Information:**

The online version contains supplementary material available at 10.1186/s12915-023-01623-0.

## Background

The NanoSIMS ion microprobe instrument is an ultra-high-resolution isotope microscope that produces quantifiable maps of chemical and isotopic variations in a wide range of materials with a lateral resolution of around 100 nm [1–3]. Technically speaking, the NanoSIMS instrument is a double-focusing, magnetic sector, multi-collection ion microprobe operating in dynamic mode, i.e., with a continuous “primary” ion-beam bombarding a flat sample surface. The impacts of these primary ions create a collision cascade in the upper atomic layers of the sample, which leads to the ejection of secondary ions (either single atoms or small molecules) from the sample surface. The secondary ions are then transmitted through the magnetic sector mass spectrometer that resolves specific isotopes from isobaric interferences in the mass spectrum. In the multi-collector, the arrivals of up to seven specific isotopes are counted in parallel by ion detectors; either electron multipliers or Faraday cups. The unique strength of the NanoSIMS ion microprobe is the ability to focus a relatively weak primary beam (order of pA; either Cs^+^ or O^−^) onto an extremely small spot, on the order of 100 nm, on the sample surface. A controlled raster of this highly focused primary beam across the sample surface can therefore, with proper standard materials as reference, provide quantitative images of chemical or isotope ratios with a lateral resolution permitting structures larger than a few hundred nanometers to be clearly resolved [1–3].

Since its commercialization about 20 years ago, the NanoSIMS instrument has been employed in numerous scientific studies across a very broad range of disciplines to produce stable isotope abundance maps in solid samples, typically when high spatial resolution is required to resolve sub-micrometer structures with modest analytical precision. It is therefore a powerful analytical instrument, e.g., in conjunction with isotope labeling experiments and characterization of biological tissue. The high spatial resolution offered by the NanoSIMS permits precise correlation of the isotope images (and quantified isotope ratio images) with transmission- and scanning electron microscopy images (i.e., TEM and SEM) as well as fluorescent microscopy, which allow specific cellular ultrastructural components to be identified based on their morphology and/or staining properties [4, 5]. These analytical capabilities have created vigorous research programs and many important biological insights have been gained across an impressive range of organisms and environments. A representative, but far from exhaustive, list of applications, with a discussion of sample preparation requirements, as well as techniques complementary to NanoSIMS imaging, can be found in Lechene et al. [2], Hoppe et al. [3], Decelle et al. [6], and Nuñez et al. [7].

The conventional NanoSIMS instrument operates at room temperature with the sample in the analysis chamber under ultra-high vacuum, in the range between 10^−9^ and 10^−10^ mbar. In order to maintain this operational vacuum, biological tissue (and other types of hydrated materials) must be prepared to minimize volatilization or degassing. Furthermore, imaging with a lateral resolution of around 100 nm requires an immobile sample with a flat surface that maintains structural integrity during analysis. As these constraints for sample preparation are similar to those required for electron microscopy, the “conventional” sample preparation procedures developed for TEM and SEM imaging are frequently employed for NanoSIMS analysis [7]. However, these procedures involve steps such as chemical fixation of the tissue (typically a mixture of (para)formaldehyde and/or glutaraldehyde), optional staining for contrast (e.g., with OsO_4_ or uranyl-acetate), and dehydration with an organic solvent (often ethanol) before embedding into a hydrophobic epoxy resin. These multiple steps are known to result in artifacts, e.g., by effectively removing soluble compounds that are not crosslinked by aldehydes and are lost from the tissue during the dehydration/embedding process [8]. Closely related to this, the process induces alterations of the original tissue morphology due to the complete removal of water, which causes shrinkage, distortion, and damage depending on the original water content of the cells and the strength of cell membranes [9]. However, macromolecular structures crosslinking with aldehydes, including many proteins, lipids, RNA, and DNA, are preserved in the sample. These macromolecular structures can be isotopically imaged with a conventional NanoSIMS instrument with subcellular resolution. Nevertheless, the isotopic compositions in the tissue under study might be dramatically affected by these sample preparation procedures. For example, the ^13^C/^12^C isotope ratios in ^13^C labeled samples can be lowered by about one order of magnitude due to dilution by C with “natural” isotope abundance ratio during resin embedding [10–12].

During the 1990s, cryofixation and cryo-substitution became available for biological samples. While such approaches avoid the usage of aldehydes, they still rely on dehydration and resin embedding at low temperature [13, 14]. Recent sample preparation developments have focused on methods that avoid ethanol dehydration and resin embedding [6, 7, 15]. For example, Loussert-Fonta et al. [12] adapted the Tokuyasu method [16] to NanoSIMS applications with a process involving weak chemical fixation of a biological tissue, cryo-protection in high-concentration sucrose, plunge freezing, cryo-sectioning, thawing, and air-drying under a thin film of polyvinyl alcohol (to preserve tissue integrity). This avoids dehydration and resin embedding altogether and still permits correlated EM and NanoSIMS imaging (as well as optical microscopy) of the same tissue section. However, even with such an approach, most soluble compounds are either greatly displaced within the cells (e.g., during thawing), or completely lost.

In order to achieve the ultimate goal of preserving a pristine tissue structure and retaining all soluble compounds in a biological tissue for correlated ultrastructure and isotopic imaging, there is no known alternative to an entirely cryogenic sample preparation and analysis workflow [17–21]. In other words, a process is needed that comprises (1) vitrification of the sample by either high pressure freezing (HPF) or plunge freezing that transforms the liquid contents of the tissue into amorphous ice, (2) surface cryo-planing to expose a particular area within the vitrified sample, (3) cryo-SEM imaging to map tissue-level and sub-cellular structures in this area of interest, and (4) high-resolution isotope imaging of the same sample surface.

The first steps in this chain have already been developed for cryo-electron microscopy of vitreous thin-sections under the name CEMOVIS; after cryo-fixation, a section ca. 200 nm in thickness is cut from the sample with a cryo-ultramicrotome and deposited on a grid before being observed in a cryo-compatible transmission electron microscope [22, 23]. However, an instrument combining ultra-high image and mass resolution with analytical sensitivity/precision permitting isotope imaging of vitrified biological tissue (or another hydrated material) at the ultrastructural level has been missing so far. Here, we report the successful development of an existing NanoSIMS instrument into an instrument that meets these analytical requirements and hence completes this cryogenic sample preparation and analytical chain. We refer to this new capability as the CryoNanoSIMS.

## Results

### The CryoNanoSIMS instrument

**Fig. 1:**
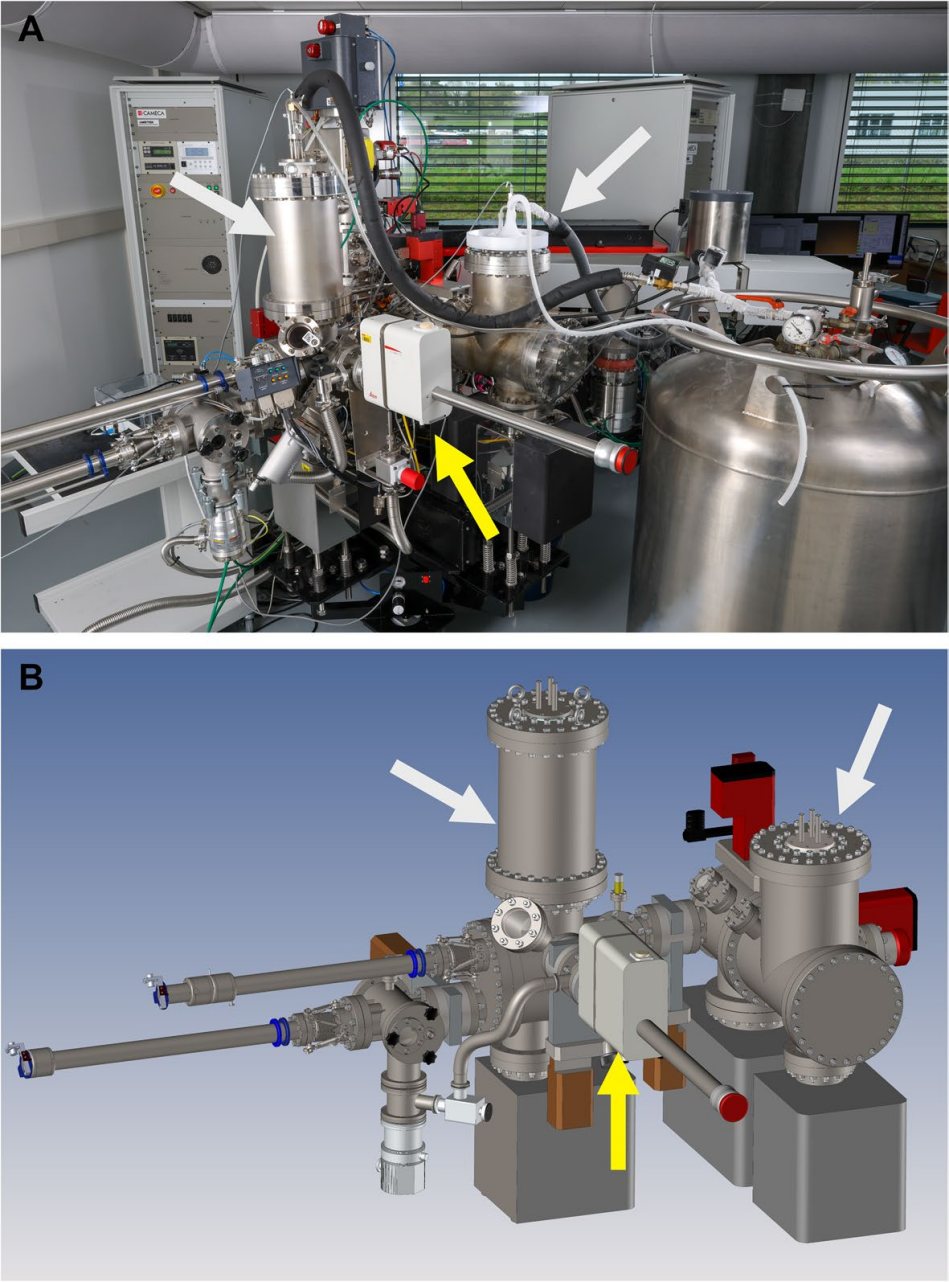
The CryoNanoSIMS. **A** Compared to the conventional NanoSIMS instrument, the analysis chamber and the sample transfer system have been redesigned to allow for the introduction of high pressure frozen, cryo-planed block-face samples with (optional) prior imaging by cryo-SEM. Notice the two liquid N_2_ reservoirs on the redesigned analysis chamber and transfer system (indicated by white arrows), both connected to the large external tank containing liquid N_2_. Also shown is the sample vacuum and cryo transfer (VCT) shuttle docked onto the transfer system (yellow arrow). **B** 3D rendering of the modified CryoNanoSIMS transfer system and analysis chamber. The CryoNanoSIMS transfer system and analysis chamber are compared with those of the conventional NanoSIMS instrument in Additional file 1: Figure S1

The CryoNanoSIMS instrument is shown in Fig. 1 during operation, i.e., with a large tank containing liquid N_2_ connected to — and hence cooling — the sample stage inside the redesigned analysis chamber as well as a sample holding position inside the cryo-sample transfer system. Through an extra port, this transfer system allows the introduction of cryogenic samples using a vacuum and cryo transfer (VCT) shuttle, in our case the Leica EM VCT500, which is seen attached to the transfer system in Fig. 1 and Additional file 1: Fig. S1. (The EM VCT500 is also compatible with an associated Gemini 500 cryo-SEM (not shown) in which samples can be imaged prior to CryoNanoSIMS isotope imaging). At the same time, this new CryoNanoSIMS transfer system maintains the capability to introduce normal room-temperature samples for conventional NanoSIMS imaging. In this incarnation, the instrument can be transformed from conventional NanoSIMS to CryoNanoSIMS simply by attaching the liquid N_2_ tank and cooling the transfer system and analysis chamber; it typically takes 10 h to complete this cooling process (Additional file 1: Fig. S2).

**Fig. 2:**
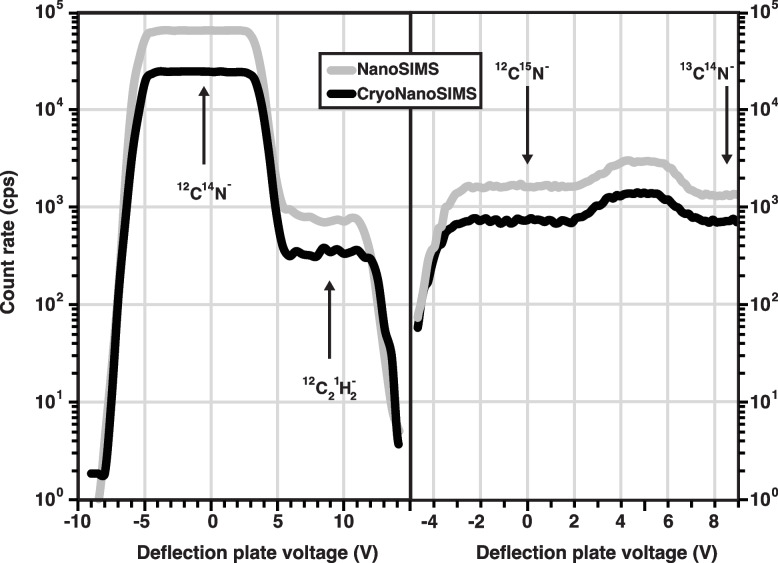
Mass-resolution for NanoSIMS *vs.* CryoNanoSIMS. Shown are mass spectra around atomic nominal mass units 26 and 27 at which the ^12^C^14^N^−^ and ^12^C^15^N^−^ ions are routinely separated from potential mass interferences and counted in electron multiplier detectors to permit quantitative measurements of the sample ^15^N/^14^N isotope ratio. In this example, the ^12^C^14^N^−^ and ^12^C^15^N^−^ ions were derived from ^15^N-labeled biological tissue (Green Hydra, cf. main text), conventionally prepared (i.e., with resin embedding) in the case of NanoSIMS analysis and with the cryogenic workflow (cf. main text) in the case of CryoNanoSIMS analysis. With identical instrument settings, the mass resolution is — for all practical purposes — indistinguishable between NanoSIMS and CryoNanoSIMS analysis. The count rates shown for each analytical mode are representative of biological tissue under typical analysis conditions, i.e., with a primary Cs^+^ beam current of ca. 2 pA focused to a spot of about 150 nm and scanning a field of view 40 × 40 µm^2^ in steps of 256 by 256 pixels; c.f. Methods. All else being equal, the count rate of ^12^C^14^N^−^ is generally lower from a vitrified sample than from a resin-embedded sample, but this can vary among different tissue types. Additional file 1: Figures S4 and S5 show the stability of key molecular ion species, isotope ratios, and the corresponding ion images obtained on isotopically normal biological tissue; see also Fig. 4

The CryoNanoSIMS operates with the sample stage held at a stable temperature of 106.2 ± 0.1 K (1 standard deviation) (Additional file 1: Fig S2). This temperature is measured by a thermocouple on the sample stage a few millimeters from the sample, which is estimated to be at around 110 K. In combination with a pressure of 1–2 × 10^−10^ mbar in the analysis chamber, these conditions ensure that ice-recrystallization and sublimation of the vitrified sample as well as surface contamination are minimized and therefore do not affect even the longest analysis, which can last many hours. Cryo-conditions are maintained by an automated re-filling of the liquid N_2_ reservoirs on the instrument (Fig. 1). The large external liquid N_2_ tank can be replaced between these automated re-fillings to assure uninterrupted cooling for as long as required (Additional file 1: Fig. S2). In this condition, the CryoNanoSIMS operates to the same technical specifications as a normal NanoSIMS with respect to all key parameters, including secondary ion transmission, mass resolution, and image resolution. Typical mass-spectra for nominal mass 26 (^12^C^14^N^−^) and 27 (^12^C^15^N^−^ with ^13^C^14^N^−^ to its right) are shown in Fig. 2 for NanoSIMS and CryoNanoSIMS analysis with identical instrument tuning (i.e., primary beam current, aperture sizes, and detector settings). The mass-resolving power of the CryoNanoSIMS is the same as that of the conventional, room-temperature instrument.

### From high pressure freezing to CryoNanoSIMS — the workflow

**Fig. 3:**
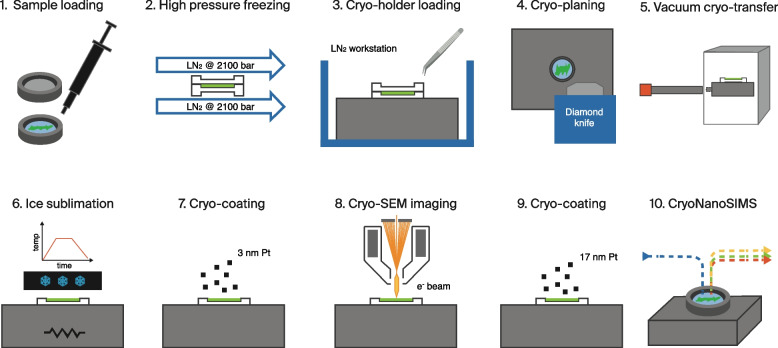
Schematic representation of the entirely cryogenic sample preparation and imaging workflow for correlated cryo-SEM and CryoNanoSIMS imaging

The entirely cryogenic sample preparation workflow, i.e., from HPF of a biological tissue (or another hydrated material) to the final CryoNanoSIMS image is schematically illustrated in Fig. 3; cf. Material & Methods for further technical details. In brief, this workflow consists of the following steps: *Step 1:* The biological material of interest is placed in a standard 3 mm diameter Au-coated Cu-carrier that is 200 μm deep. Any remaining volume of the carrier is filled with cryoprotectant solution in part to avoid trapped air that can prevent vitrification; we have found that dextran 40 produces the best overall results with respect to cryo-ultra-microtome trimming and planning (see below). Then the Cu-carrier is sealed with the flat side of an Al-carrier of the same dimensions; this operation takes place in a matter of seconds directly on a HPF instrument. *Step 2*: The loaded Al-Cu carrier sandwich is immediately vitrified in a HPF instrument after which it drops into a bath of liquid N_2_, in which it can be stored as long as needed. *Step 3*: The carrier sandwich containing the vitrified sample is fixed in a sample holder compatible with all the instruments to be employed subsequently and the Al-lid is removed; this takes place in a controlled liquid nitrogen bath system (cryo-workstation). *Step 4*: The sample in the Cu-carrier is planed with a cryo-ultramicrotome using a diamond knife, creating a flat block-face surface (Additional file 1: Fig. S3). *Step 5*: The holder is moved with the VCT shuttle to a cryo metal-coater. *Step 6*: Sublimation of surface ice is performed under vacuum in the cryo metal-coater in order to remove any surface-ice contamination and create a controlled surface relief that reveals the ultrastructure of biological tissue [24]. *Step 7*: Subsequently, a layer of ~ 3 nm Pt is deposited with an e-beam evaporator for surface conductivity. *Step 8*: The sample is transferred in the VCT shuttle to a cryo-SEM for surface imaging. *Step 9*: The sample is transferred back to the cryo metal-coater for the deposition of an additional ~ 17 nm of Pt. *Step 10*: The sample is transferred in the VCT shuttle to the CryoNanoSIMS and isotopically imaged.

### Correlated cryo-SEM and CryoNanoSIMS versus conventional SEM and NanoSIMS imaging

**Fig. 4:**
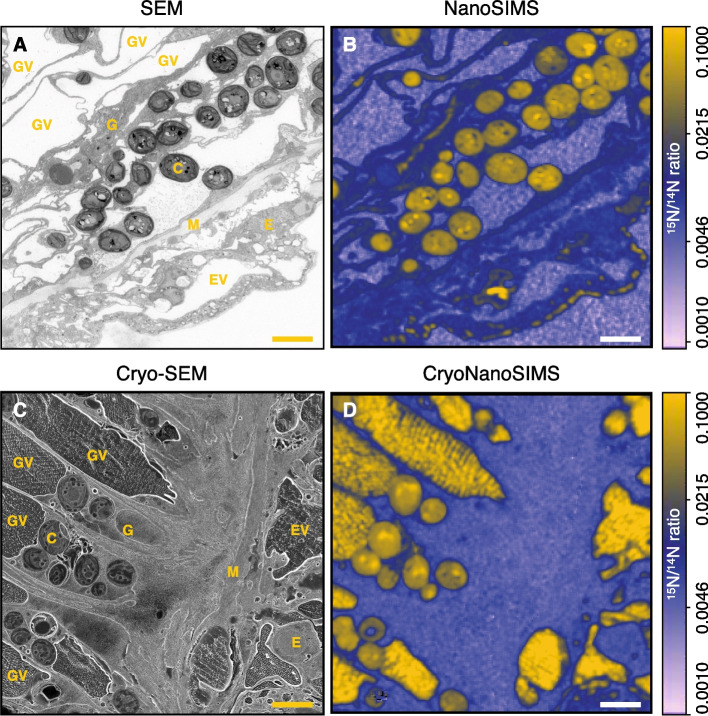
Correlated SEM and NanoSIMS isotope ratio images for room-temperature and cryogenic workflows. The tissue imaged was from the symbiotic freshwater organism Green Hydra, isotopically labeled through the assimilation of ^15^NH_4_^+^. **A** SEM image of a section of conventionally prepared tissue and **B** a quantified NanoSIMS map (room-temperature) of the corresponding ^15^N/^14^N ratio. **C** Cryo-SEM image after the entirely cryogenic sample preparation and **D** a quantified CryoNanoSIMS map of the corresponding ^15^N/^14^N ratio. The images were obtained from similar regions in the gastric region of Green Hydra animals that were exposed to identical incubation conditions (cf. Methods), permitting a direct comparison between the two analytical approaches. Note the dramatic difference in appearance between conventionally prepared and cryogenically prepared tissue (**A** *vs.* **C**), i.e., shrinkage and strong deformation (e.g., of vacuoles) *vs.* no shrinkage/deformation, and the strong ^15^N enrichments in the vacuoles in the CryoNanoSIMS image, which are absent in the (emptied) vacuoles after normal sample preparation (**B** *vs*. **D**). The isotopic enrichments shown in **B** and **D** are presented by a logarithmic color scale. The isotope ratio images are drift-corrected accumulations of 5 sequential images each consisting of 256 by 256 pixels with a pixel dwelling time of 5 ms; typical analysis time for one image as displayed is about 30 min. Scale bars are 5 μm. C, *Chlorella* sp. algae; EV, epidermal vacuole; E, epidermis; GV, gastrodermal vacuole; G, gastrodermis; M, mesoglea

The results of the correlated cryo-SEM and CryoNanoSIMS imaging, prepared according to the entirely cryogenic sample preparation workflow described above, can be directly compared with room temperature SEM and NanoSIMS imaging of similar tissue, derived from the same isotopic labeling experiment but prepared with a conventional sample preparation protocol (Fig. 4). Here, we used Green Hydra (*Hydra viridissima*) to draw this comparison. This freshwater hydrozoan hosts a population of endosymbiotic green algae (*Chloeralla *sp.) contained in symbiosomes inside its gastrodermal cells [25]. Specimens of Green Hydra were incubated for 6 h with 20 µM of ^15^NH_4_^+^ (a limiting macronutrient) under illumination; cf. Methods. This incubation permitted the uptake and assimilation of ^15^NH_4_^+^ by both the Green Hydra host and its symbiotic algae, creating contrast in the ^15^N/^14^N ratio inside the tissue that is easily imaged with conventional NanoSIMS workflow, as it was done in previous studies of, e.g., cnidarian-algal interactions [26–30]. Similar regions in the gastric tissue of these Green Hydra animals were targeted during sample preparation for NanoSIMS and CryoNanoSIMS imaging in order to facilitate direct comparison.

As expected, the tissue prepared with conventional EM protocols (Fig. 4A) had an appearance radically different from similar tissue prepared with the cryogenic workflow described above (Fig. 4C). Conventional tissue preparation created well-known artifacts, in particular, strong shrinkage due to the dehydration steps. Hydrated and gel-like tissue structures, such as vacuoles initially containing liquid or the gel-like mesoglea were strongly diminished and/or deformed. In contrast, these same tissue structures appeared full of their natural contents and in their original shapes after HPF and subsequent cryo-preparation (Fig. 4C), compared with in vivo imaging [31].

Equally striking was the comparison between the resulting NanoSIMS and CryoNanoSIMS images (Fig. 4B versus Fig. 4D). Bombarding these samples with a focused Cs^+^ beam to create images of the ^15^N/^14^N ratio (cf. Methods), the symbiotic algae were shown to be strongly labeled regardless of sample preparation, because ^15^N originally taken up by the host as ^15^NH_4_^+^ was incorporated into structural components of these cells through anabolic pathways, and these cellular structures were not significantly altered during conventional sample preparation [27–29]. In contrast, vacuoles in the gastrodermal and epidermal cell layers, which had lost all their original content in conventional sample preparation and thus appeared unlabeled in ^15^N (Fig. 4B), were still full of their original liquid content and appeared strongly enriched in ^15^N in CryoNanoSIMS images (Fig. 4D).

**Fig. 5:**
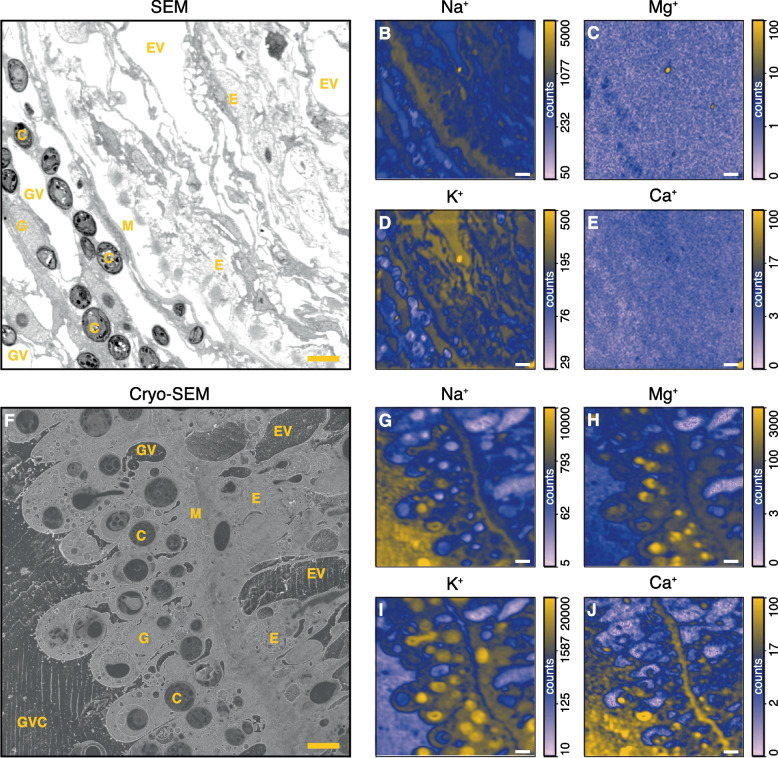
Correlated SEM and NanoSIMS inorganic ion images for room-temperature and cryogenic workflows. **A** SEM image of a section of conventionally prepared tissue of Green Hydra and corresponding NanoSIMS maps of the distributions of Na (**B**), Mg (**C**), K (**D**), and Ca (**E**). **F** Cryo-SEM image after a fully cryogenic sample preparation of tissue of Green Hydra and corresponding CryoNanoSIMS maps of the distributions of Na (**G**), Mg (**H**), K (**I**), and Ca (**J**). The images are from similar regions in the gastric region of Green Hydra animals that were exposed to identical incubation conditions (cf. Methods), permitting a direct comparison between the two analytical approaches. Note the dramatic loss of inorganic ions (especially Mg, K, and Ca) in conventional sample preparation due to dehydration and resin infiltration of the samples. Elemental maps are shown in a logarithmic color scale. These elemental maps are drift-corrected accumulations of 15 sequential images each consisting of 256 by 256 pixels with a pixel dwelling time of 5 ms; typical analysis time for one image as displayed is about 90 min. Scale bars are 5 µm. C, *Chlorella* sp. algae; EV, epidermal vacuole; E, epidermis; GVC, gastrovascular cavity; GV, gastrodermal vacuole; G, gastrodermis; M, mesoglea

Bombarding adjacent regions in the same samples with a focused O^−^ beam permitted to visualize key trace elements critical to cellular homeostasis and osmoregulation; here Na, Mg, K, and Ca (Fig. 5). The concentrations of these elements were not manipulated in the medium prior to sample preparation. In the direct comparison between the conventional NanoSIMS images and the CryoNanopSIMS images, several important observations were made. First, with conventional sample preparation, Mg and Ca were essentially completely lost from the tissue compared with cryo-preparation. But, CryoNanoSIMS imaging demonstrated that these elements were originally concentrated in the algal endosymbionts/symbiosomes and in the mesoglea, respectively (Fig. 5C,E *vs*. H,J).

Na and K also appeared partly lost from the tissue in conventional preparation. But, of equal importance, although not completely removed from the tissue it was evident that K was dramatically redistributed during conventional sample preparation, compared with the fully cryo-genic workflow (Fig. 5B,D *vs*. G,I). For example, the algal symbionts were rich in K in the CryoNanoSIMS image(s) (Fig. 5I), but exhibited relatively low content of K in the conventional NanoSIMS image(s) (Fig. 5D). Such images of cellular level distributions of Na, Mg, K, and Ca provide a direct visualization of the highly dynamic and controlled distribution of cations that is essential to the homeostasis of multicellular organisms [32].

It is noted that, following CryoNanoSIMS imaging as described here, the sample surface did not exhibit signs of melting. Cryo-SEM images of a typical sample surface before and after CryoNanoSIMS analysis are shown in Additional file 1: Fig. S6.

## Discussion

Correlated cryo-SEM and CryoNanoSIMS imaging opens vast new research horizons. With this technological development, it is now possible to visualize and quantify the uptake and/or cellular-level distribution of a wide range of soluble elements/molecules (e.g., nutrients or drugs) in any biological tissue or other hydrated material with a sub-micrometer lateral resolution, provided that their distribution inside the sample creates distinct isotopic or chemical ion-image features, and provided that the material is amenable to HPF (Figs. 4 and 5). A major advantage is the absence of artifacts, in particular tissue shrinkage and loss of soluble compounds, which — to different degrees — cannot be avoided except with an entirely cryogenic workflow (Fig. 3).

To illustrate the unique possibilities of CryoNanoSIMS, the uptake and assimilation of ^15^NH_4_^+^ in the *Hydra*-*Chlorella* symbiosis were visualized. Consistent with previous conventional NanoSIMS studies of ^15^NH_4_^+^ assimilation in marine cnidarians [27], we found that *Hydra* epithelial and gastrodermal cells, as well as their intracellular algal symbionts, became distinctly enriched in ^15^N. While this is evident from both conventional and cryogenic NanoSIMS imaging workflows (Fig. 4), only the cryo-imaging approach maintained an undistorted morphology of the tissue.

Equally important, the CryoNanoSIMS images revealed that epithelial and gastrodermal vacuoles showed the highest ^15^N enrichment within the *Hydra* tissue as a result of ^15^NH_4_^+^ assimilation (Fig. 4D). As these vacuoles are not connected to the external environment of the animal, this enrichment is unlikely to be the result of direct exchange with the surrounding medium, i.e., it requires active transport [33]. At the same time, the high ^15^N enrichment observed in these vacuoles indicates that they also play a role in the N metabolism of *Hydra*. Specifically, these vacuoles might be facilitating efficient excretion of excess inorganic N from the host cells, thereby contributing to the removal of metabolic waste products and maintaining N-limited conditions in the tissue, which is thought to be a requirement for a stable symbiosis with the algal symbionts [34–37].

In addition, it has previously been hypothesized that these vacuoles play an important role in the osmoregulation of *Hydra* by excreting sodium into the gastrovascular cavity while retaining potassium within the animal cells [33, 38, 39]. The CryoNanoSIMS images in Fig. 5G–J visualize the natural subcellular distribution of these elements and indeed show a relatively high sodium content in host gastrodermal vacuoles, the mesoglea, and the gastrovascular cavity and a relatively high K content in the host and symbiotic algal cells, in clear support of the osmoregulation hypothesis [33].

The images shown in Figs. 4 and 5 demonstrate the strong advantages of the CryoNanoSIMS for the characterization of fluid and solute dynamics in biological tissues, or other hydrous samples. Note that the impact of the electron beam during cryo-SEM imaging does not produce any noticeable effects on subsequent CryoNanoSIMS isotopic mapping, judging from isotopic ratio maps obtained on both isotopically normal (control) and isotopically enriched (and hence more heterogeneous) samples. Note also the presence of a certain level of freezing artifacts in the large vacuoles, as well as in the gastrovascular cavity in Figs. 4 and 5, in the form of linear crystallization features that become visible after sublimation (*Step 6* in Fig. 3). The absence of cryoprotectant within these enclosed aqueous compartments likely contributes to this and enhances devitrification. These linear features also appear in the CryoNanoSIMS ^15^N/^14^N maps (Fig. 4), which might represent a mild “edge-effect” that slightly fractionates the measured ^12^C^15^N^−^/^12^C^14^N^−^ ratio. However, similar topography in other parts of the tissue did not result in similar isotopic effects on isotopically normal control samples. In any case, this does not affect the conclusions drawn above and future improvement of the HPF process and/or slightly less sublimation might alleviate this artifact.

In general, for the cryogenic workflow described above (Fig. 3), a practical constraint to consider is that the sample has to fit into a sample carrier for HPF, as schematically illustrated in Fig. 3 (*Step 1*). These carriers are 3 or 6 mm in diameter and can contain samples up to 300 μm thick. Samples larger than these dimensions must be cut to fit into the carrier. In general, in order to obtain high-quality HPF results, a biological tissue should not exceed about 200 μm in thickness; thicker samples will not freeze fast enough in their interior to avoid ice crystal formation and associated tissue damage [40, 41]. However, within these constraints, it is possible to achieve quality HPF of a wide range of tissues and other hydrated materials [42].

With regard to the detection of specific isotopes or isotopically labeled soluble molecules, there are several aspects to consider. First, in a dynamic SIMS ion microprobe, such as the NanoSIMS, the primary ion beam hitting the sample creates ions from the material involved in the collision cascade under the beam. These collisions generally break larger molecules into smaller fragments and chemical information is thus lost [43]. Second, the isotopic label has to make the molecule or the element in question distinct from other sample constituents. This is often achieved through experiments that enrich the element or molecule of interest in a low abundance stable isotope, e.g., ^13^C, ^15^N, ^18^O, ^26^Mg, ^34^S, and ^44^Ca. At a given mass in the mass spectrum of emitted secondary ions, the ions reaching the detectors will be a mixture of experimentally added and naturally present isotopes. In order to detect an isotopic enrichment in the sample, it is therefore required that the experimentally added isotopes or labeled molecules are present in concentrations high enough to alter the chemistry or isotopic composition of the tissue measurably. The minimum concentration at which an isotope or labeled molecule becomes detectable will depend not only on the chemical composition of the sample itself but also on the ionization yield of the secondary ion in question. It is hard to predict a priori what the detection limit of a given element or molecule will be. We are currently in the process of exploring a range of different biological tissues that have assimilated various isotopically labeled compounds and elements, to generate a better understanding of the experimental opportunities generated by the CryoNanoSIMS instrument.

However, it is clear that, in the many situations in which an isotope or molecule can be detected in a biological (or another hydrated) material that is amenable to HPF, the correlated cryo-SEM and CryoNanoSIMS imaging capabilities presented here permit fundamentally important research questions to be addressed in an entirely new manner.

## Conclusion

By avoiding artifacts associated with existing NanoSIMS sample preparation methods, a fully cryogenic workflow made possible by the CryoNanoSIMS instrument permits sub-cellular isotope and elemental mapping of tissues in their natural ultrastructural configurations, without the loss or displacement of soluble compounds. These novel capabilities will have a strong and broad impact in the fields of, e.g., environmental science, biology, and related life sciences, as well as material science.

## Methods

### Biological material and experimental protocol

Specimens of Green Hydra strain A99 were reared in a 12 h:12 h light/dark cycle at 20 °C in clear food containers filled with 1 L M-solution [44] using an Algaetron 230 incubator (Photon System Instruments). Once per week, animals were fed with freshly hatched *Artemia nauplii*, the containers were cleaned, and the M-solution was replaced. For isotope labeling, animals were starved for 1 week, before being transferred into individual 10 ml glass vials filled with M-solution containing 20 µM ^15^NH_4_Cl (> 98 atom %, Sigma-Aldrich) and incubated in the light (50 µE m^−2^ s^−1^) at 20 °C for 6 h. Following incubation, half of the animals were immediately fixed for conventional NanoSIMS imaging, whereas the other half was immediately high pressure frozen for correlated cryo-SEM and CryoNanoSIMS imaging.

### Sample preparation for room temperature SEM and NanoSIMS imaging

For initial fixation, animals were transferred into fixative (1.25% glutaraldehyde and 0.5% paraformaldehyde in 0.1 M phosphate buffer) and incubated for 1 h at room temperature followed by another 24 h at 4 °C. Animals were rinsed in three washes of PBS (1X) and stained in osmium tetroxide solution (1% in ddH_2_O) for 1 h at 4 °C. Following three washes in MiliQ water, the samples were dehydrated in an ethanol series of increasing concentrations (10 min in 30%, 10 min in 50%, 2 × 10 min in 70%, 3 × 10 min in 90%, 3 × 10 min 100%), transferred to acetone (10 min in 100%) to ensure better miscibility with resin and gradually infiltrated with SPURR epoxy resin (Spurr’s Low Viscosity Embedding Media Kit, Electron Microscopy Sciences) in increasing concentration (30 min in 25%, 30 min in 50%, 1 h in 75%, overnight in 100%). Following infiltration, animals were embedded in bottleneck tip embedding capsules (BEEM) in fresh SPURR resin at 65 °C for 48 h. Subsequently, 200 nm thin sections were cut (Ultracut E ultra-microtome; Reichert) and transferred on glow-discharged silicon wafers. Sections on wafers were stained with 1% uranyl acetate (Electron Microscopy Sciences) in water for 10 min, followed by Reynolds lead citrate solution (Electron Microscopy Sciences) for 10 min. Images were taken on a GeminiSEM 500 field emission scanning electron microscope (Zeiss) at 3 kV with an aperture size of 30 µm, and a working distance of 2.2 mm, using the energy selective backscatter detector (EsB; ZEISS) with the filter-grid set at 121 V.

### Sample preparation for correlated cryo-SEM and CryoNanoSIMS imaging

In the following, the indicated steps in the workflow correspond to the steps schematically illustrated in Fig. 3.

#### Steps 1 and 2: Sample loading and high pressure freezing

Following incubation with ^15^NH_4_^+^, animals were carefully detached and immediately transferred to type A 3 mm Au-coated Cu-carriers (large cavity, 200 μm deep, Wohlwend) filled with dextran 40 in M-solution (20% w/v), which serves as cryoprotectant and filler. Each carrier containing one animal was immediately covered with the flat side of a type B Al-carrier previously coated in 1-hexadecene. The sample was immediately high pressure frozen using an EM ICE (Leica) device, which creates an optimum pressure of 2100 bar on the sample within a few milliseconds followed by immediate rapid cooling [45]. The hexadecene coating helps to detach the type B carrier from the type A carrier after high pressure freezing without damaging the surface of the vitrified sample that remains in the type A Cu-carrier during all further steps.

#### Steps 3 and 4: Mounting and cryo-planing of the vitrified sample block-face

Two Cu-carriers with samples were loaded into a custom-built cryo sample holder in a Leica instrument EM VCM, which provided a stable cryo-environment (liquid N_2_ bath) for sample and holder manipulation. Once placed in the cryo-holder the two samples were submerged in liquid N_2_ and transferred to a Leica cryo-chamber EM FC7 on a Leica EM UC7 Ultramicrotome, previously equilibrated at a temperature of 163 K by a controlled flow of liquid N_2_. When the Al-lid was removed from the Al-Cu carrier sandwich the surface of the sample was level with the rims of the Cu-carrier, but the surface was rough. Subsequent cryo-SEM and CryoNanoSIMS imaging require a flat and smooth surface, which was obtained by planing the block with a diamond knife in the cryo-ultramicrotome. However, it is not possible to create the required surface flatness across the entire area of the 3 mm Cu-carrier. Therefore, a smaller surface was created in the center of the Cu-carrier by trimming away the carrier metal with a trimming diamond knife, keeping a square (now raised) surface containing the vitrified sample in the middle of the carrier (Additional file 1: Fig. S3). This raised surface containing the sample was smoothed with a dedicated diamond cryo TRIM20 Diatome knife.

#### Step 5: Cryo transportation

From this point on, the cryo-holder with the two Cu-carriers was moved between the different instruments using a vacuum and cryo transfer (VCT) shuttle; the Leica EM VCT500. This shuttle actively keeps the sample at a temperature below 120 K in a vacuum of 8 × 10^−4^ mbar.

#### Steps 6 and 7: Ice sublimation and coating of the samples

Using the VCT the sample was inserted into a Leica cryo-eBeam metal-coater EM ACE600, previously cooled down to a temperature of 123 K and pumped to a vacuum of 9 × 10^−7^ mbar. Samples were exposed to an ice sublimation cycle [24], during which the temperature was ramped up from 123 to 180 K at 3 K per minute and held at 180 K for 7 min, before the temperature was returned back to 123 K. This temperature cycle removes surface ice contamination and brings out ultrastructural details for subsequent cryo-SEM imaging. The sample was then coated with a ca. 3 nm layer of Pt. Afterwards, the holder was collected by the VCT shuttle and transferred to the cryo-SEM for imaging.

#### Step 8: Cryo-SEM imaging

The cryo-holder was placed in a Gemini 500 cryo-SEM at a temperature of 133.2 K with a vacuum pressure of 2.7 × 10^−8^ mbar. The samples were imaged at a working distance of 5 mm using the BSE in-lens detector and a beam energy of 1.7 kV. The aperture size was 10 µm. Images of 4098 × 3072 pixels were obtained from areas of interest within the sample.

#### Steps 9 and 10: Coating of the samples and transfer to CryoNanoSIMS

After cryo-SEM imaging, the samples were transferred back to the EM ACE600 device for thicker Pt coating in preparation for CryoNanoSIMS, using both e-beam coating columns to produce a 20 nm conformal Pt layer on the sample surface without “shaded” surface regions. After this coating, the holder was moved back to the EM VCT500 shuttle and transported to the CryoNanoSIMS.

### NanoSIMS and CryoNanoSIMS analysis parameters

The instrument settings for NanoSIMS and CryoNanoSIMS imaging were identical. In the operational mode using 16 keV Cs^+^-ions, the sample surface was first pre-sputtered with a defocused primary beam delivering a total dose of 8 × 10^14^ Cs^+^-ions per cm^2^ to remove the metal coating and approach sputtering equilibrium with the sample surface. The same sample surface was then bombarded with a primary ion beam of ca. 2 pA Cs^+^ focused to a spot size of about 150 nm. Secondary molecular cyanide ions ^12^C^14^N^−^ and ^12^C^15^N^−^ were simultaneously collected in electron multipliers at a mass resolution of about 9000 (Cameca definition), enough to resolve the ^12^C^15^N^−^ ions from potentially problematic interferences (Fig. 2). Images of 40 × 40 μm^2^ with 256 × 256 pixels were obtained by rastering the primary beam across the sample surface with a pixel dwell-time of 5 ms. Up to ten isotope images were recorded of the same sample area and ^15^N/^14^N maps were formed by taking the ratio between the drift-corrected and subsequently accumulated ^12^C^15^N^−^ and ^12^C^14^N^−^ ion images. Additional file 1: Figures S4 and S5 show typical ion count rates, beam stability, isotopic ratio stability, and ion images from a CryoNanoSIMS run on *Hydra* tissue with natural isotopic composition.

In the operational mode using 16 keV O^−^ ions, the sample surface was first pre-sputtered with a defocused primary beam delivering a total dose of about 2 × 10^16^ O^−^-ions per cm^2^ to remove the metal coating and approach sputtering equilibrium with the sample surface. The same sample surface was then bombarded with a primary beam of ca. 2 pA O^−^-ions focused to a spot size of about 350 nm. Secondary ions ^23^Na^+^, ^24^Mg^+^, ^39^K^+^, and ^40^Ca^+^ were simultaneously collected in electron multipliers at a mass resolution higher than 6000, enough to resolve any potentially problematic interferences. Images of 55 × 55 μm^2^ with 256 × 256 pixels were obtained by rastering the primary beam across the sample surface with a pixel dwell-time of 5 ms. Up to 15 isotope images were recorded of the same sample area, drift-corrected, and subsequently accumulated.

In conventional NanoSIMS mode, the pressure in the analysis chamber was about 5 × 10^−10^ mbar. In the cryo-mode, the analysis chamber pressure was typically lower, around 1–2 × 10^−10^ mbar. The electron-flood gun was not required for any of the NanoSIMS imaging presented here.

## Supplementary Information

**Additional file 1: Fig. S1.** 3D renderings comparing conventional and CryoNanoSIMS transfer systems.** Fig. S2.** Cooling curve for the CryoNanoSIMS.** Fig. S3.** Schematic drawing illustrating ultramicrotome cryo-planing.** Fig. S4.** CryoNanoSIMS analysis of carbon isotope ratios in Green Hydra.** Fig. S5.** CryoNanoSIMS analysis of nitrogen isotope ratios in Green Hydra.** Fig. S6.** SEM images of sample surfaces before and afterNanoSIMS imaging.

## Data Availability

All data are available in the main text or the supplementary materials.

## Abbreviations

HPF: High pressure freezing
NanoSIMS: Nanoscale secondary ion mass spectrometry
SEM: Scanning electron microscopy
TEM: Transmission electron microscopy
VCT: Vacuum and cryo transfer
