# Supplementary material for: Correlated cryo-SEM and CryoNanoSIMS imaging of biological tissue
Source: BMC Biol. 2023 Jun 7;21:126. doi: 10.1186/s12915-023-01623-0 (PMC10246362; doi:10.1186/s12915-023-01623-0)
Supplement: Supplementary file 1 — Additional file 1: Fig. S1. 3D renderings comparing conventional and CryoNanoSIMS transfer systems. Fig. S2. Cooling curve for the CryoNanoSIMS. Fig. S3. Schematic drawing illustrating ultramicrotome cryo-planing. Fig. S4. CryoNanoSIMS analysis of carbon isotope ratios in Green Hydra. Fig. S5. CryoNanoSIMS analysis of nitrogen isotope ratios in Green Hydra. Fig. S6. SEM images of sample surfaces before and afterNanoSIMS imaging. [file 12915_2023_1623_MOESM1_ESM.pdf]

Supplementary Materials for

Correlated Cryo-SEM and CryoNanoSIMS Imaging of Biological Tissue

Anders Meibom*, Florent Plane, Tian Cheng, Gilles Grandjean, Olivier Haldimann, Stephane Escrig, Louise Jensen, Jean Daraspe, Antonio Mucciolo, Damien De Bellis, Nils Rädecker, Cristina Martin-Olmos, Christel Genoud, and Arnaud Comment.

*Corresponding author. Email: anders.meibom@epfl.ch

This file includes:

Fig. S1: 3D renderings comparing conventional and CryoNanoSIMS transfer systems
Fig. S2: Cooling curve for the CryoNanoSIMS
Fig. S3: Schematic drawing illustrating ultramicrotome cryo-planing
Fig. S4: CryoNanoSIMS analysis of carbon isotope ratios in Green Hydra
Fig. S5: CryoNanoSIMS analysis of nitrogen isotope ratios in Green Hydra
Fig. S6: SEM images of sample surfaces before and after (Cryo-)NanoSIMS imaging

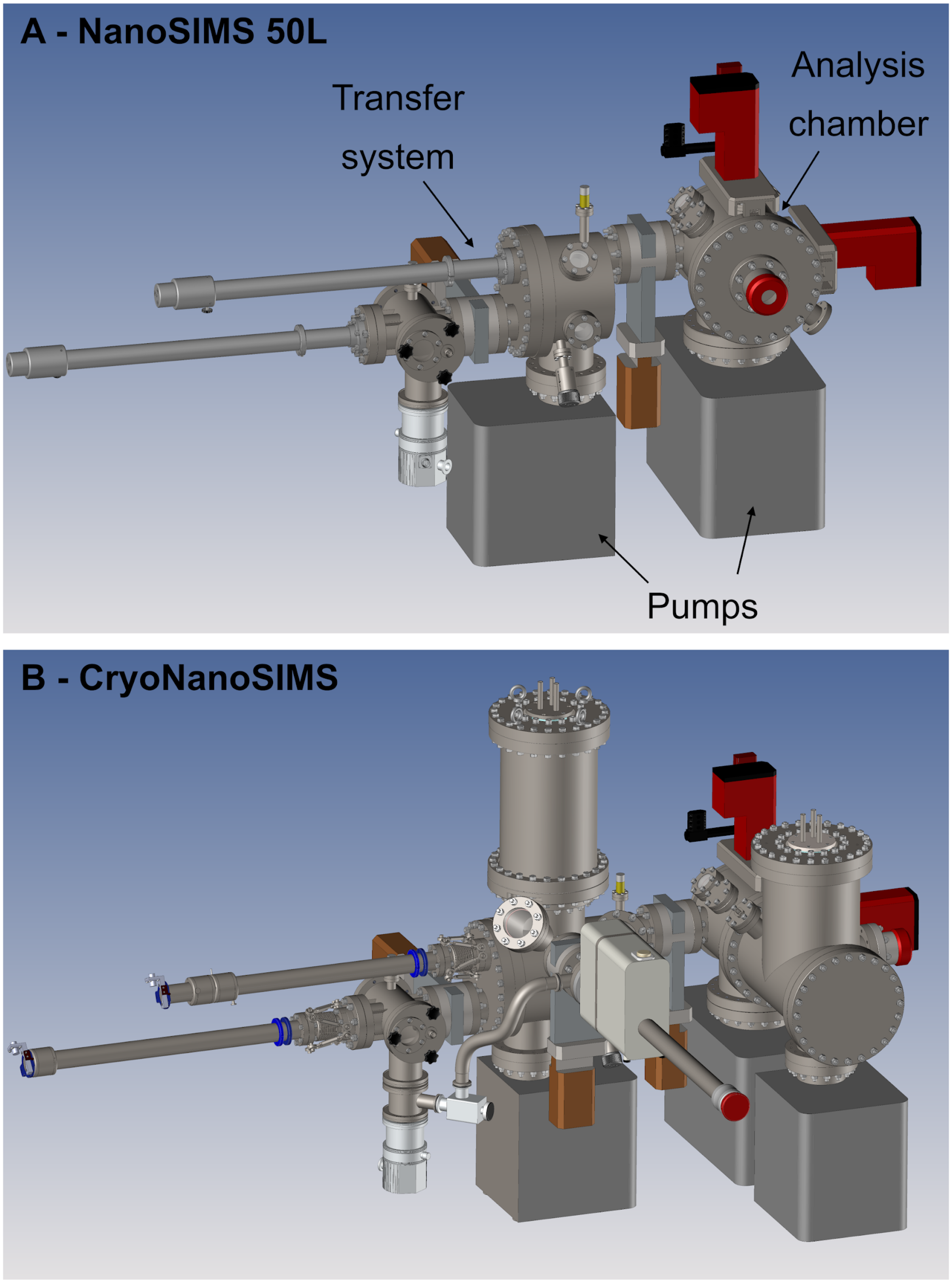

Figure S1. 3D renderings comparing the transfer system and analysis chamber of the conventional NanoSIMS instrument (**A**) with those of the CryoNanoSIMS (**B**).

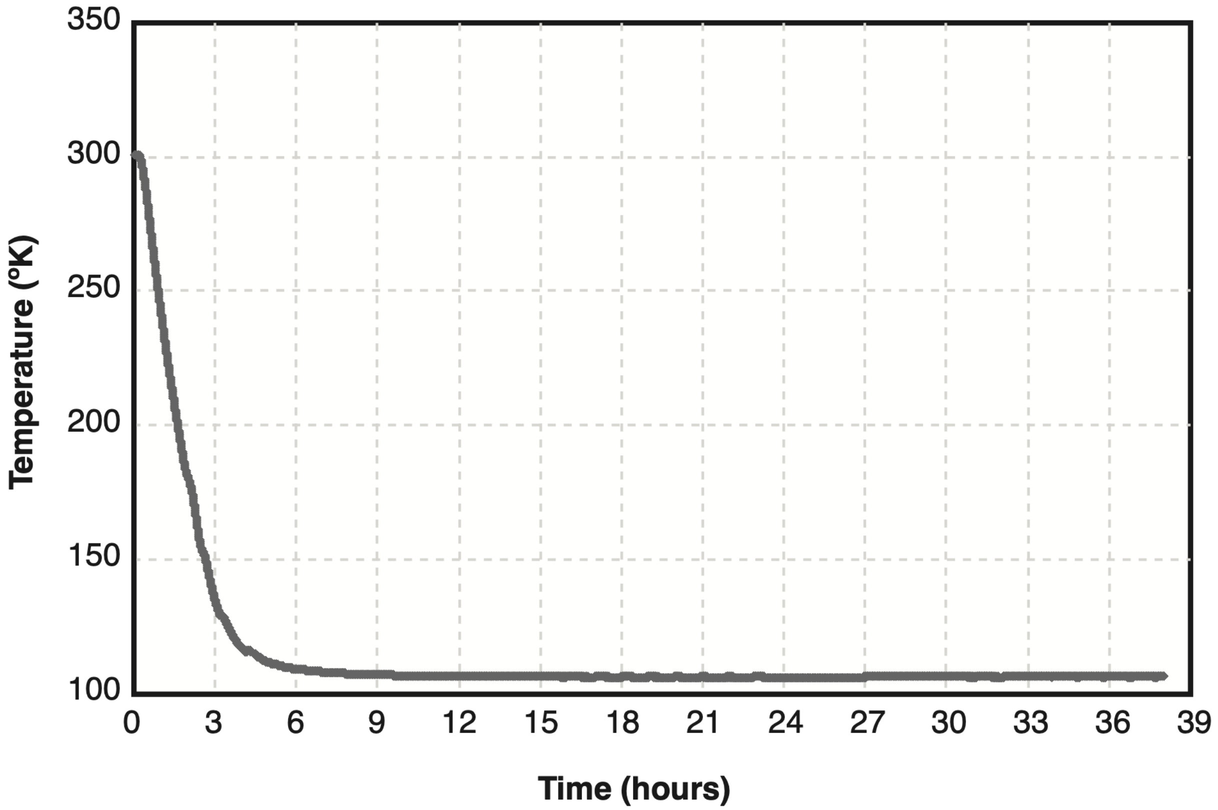

Figure S2. Cooling curve for the CryoNanoSIMS. The temperature is measured with a thermocouple on the sample stage a few mm from the sample itself. Starting at room temperature, a stable temperature of 106.2 K is reached in about 10 hours, after which the temperature variation is within ± 0.1 K (1 standard deviation) for as long as liquid N_2 is supplied to the instrument.

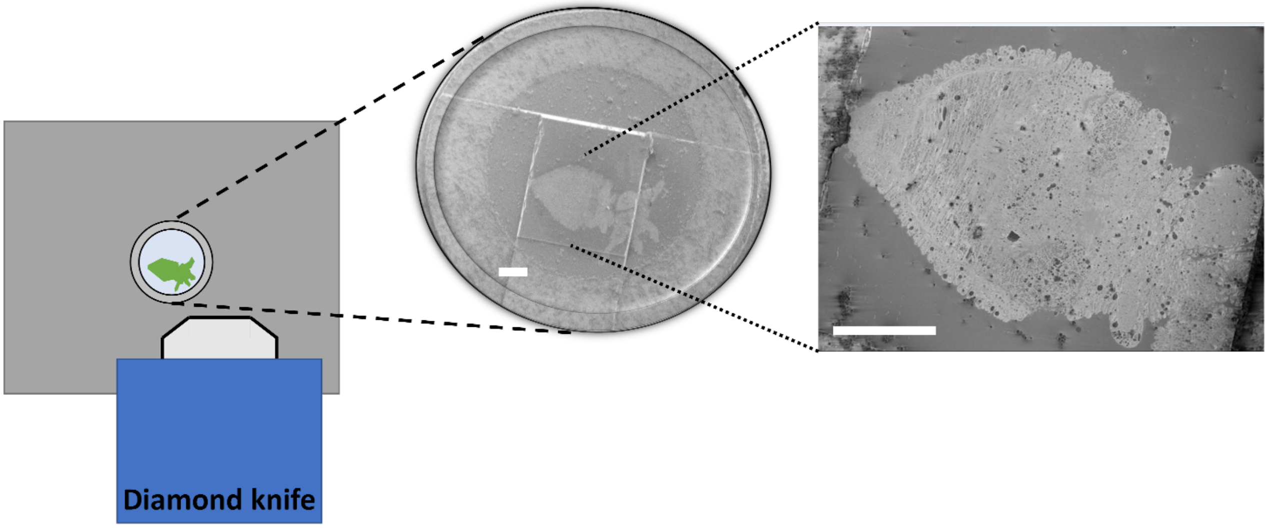

Figure S3. Schematic drawing illustrating ultramicrotome cryo-planing. The diamond knife trims and planarizes the sample (left) creating a rectangular pyramid with a very flat block-face surface as shown in the cryo-SEM image (middle). Right: Cryo-SEM micrograph of a Green Hydra sample. Scale bars are 250 µm.

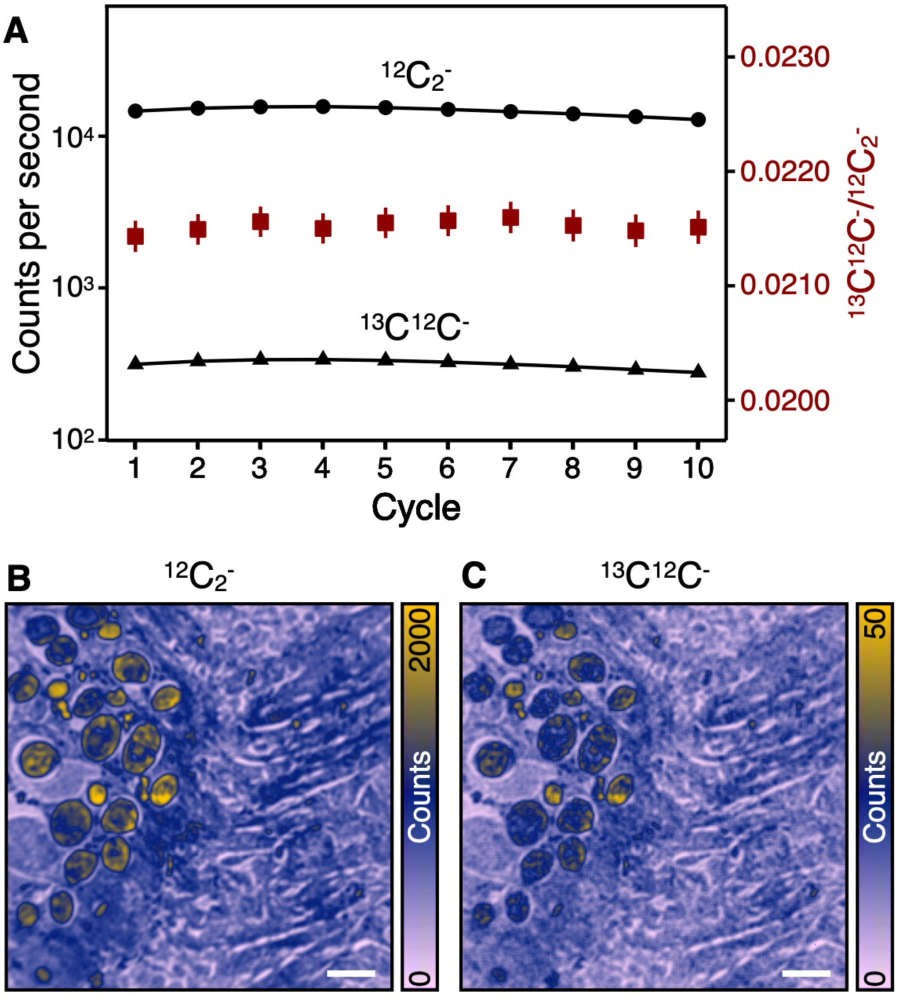

Figure S4. CryoNanoSIMS analysis of carbon isotope ratios in Green Hydra: count rates, isotope ratio, and ion images obtained from analysis of isotopically normal tissue of Green Hydra. (**A**) Average $^{12}C_2^-$ and $^{13}C^{12}C^-$ ion count rates and corresponding isotope ratio over 10 imaging cycles for the entire image. Each cycle consisted of a 256x256 pixel raster with a beam dwelling time of 5 milliseconds, for a total imaging time of ca. 60 minutes. Isotope ratio error bars are ±2 std error. (**B** and **C**) Drift-corrected and accumulated ion-maps for $^{12}C_2^-$ and $^{13}C^{12}C^-$, respectively. Scale bars are 5 µm.

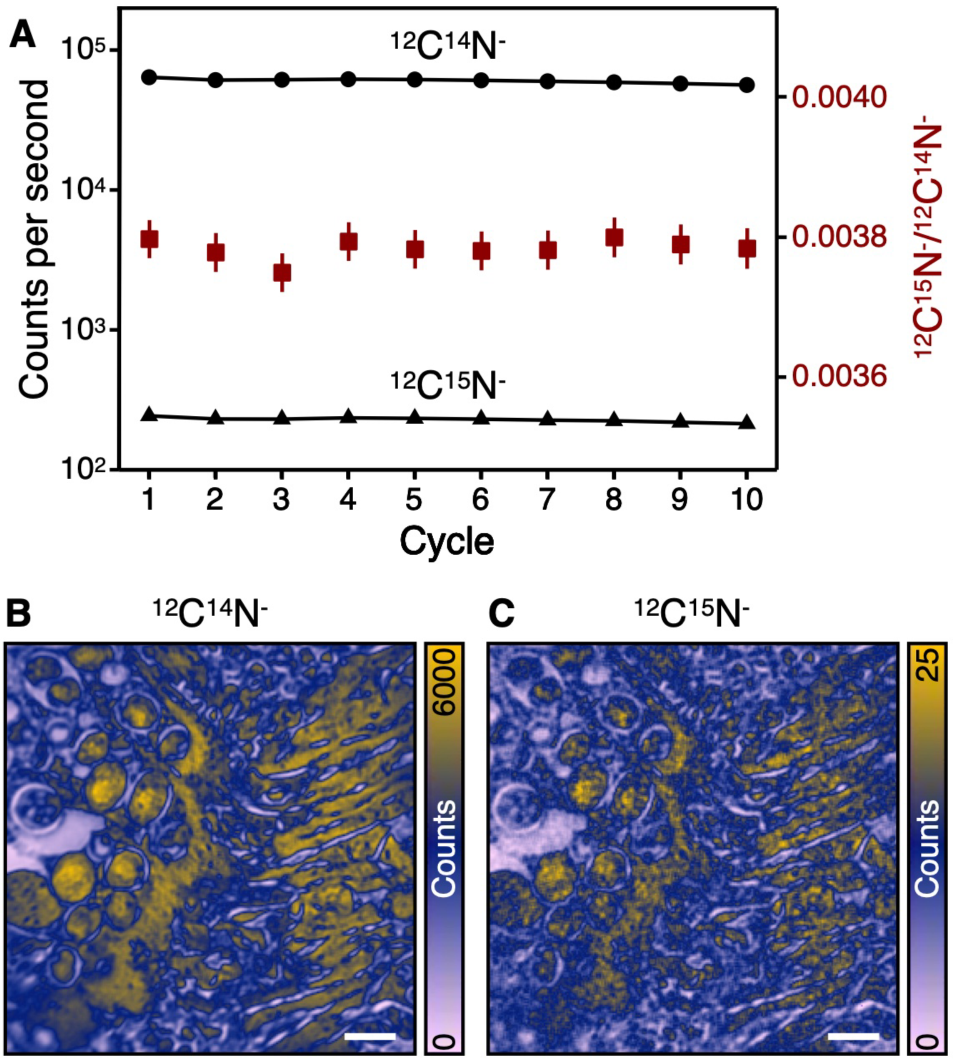

Figure S5. CryoNanoSIMS analysis of nitrogen isotope ratios in Green Hydra: count rates, isotope ratio, and ion images obtained from analysis of isotopically normal tissue of Green Hydra. (**A**) Average $^{12}C^{14}N^-$ and $^{12}C^{15}N^-$ ion count rates and corresponding isotope ratio over 10 imaging cycles for the entire image. Each cycle consisted of a 256x256 pixel raster with a beam dwelling time of 5 milliseconds, for a total imaging time of ca. 60 minutes. Isotope ratio error bars are ±2 std error. (**B** and **C**) Drift-corrected and accumulated ion-maps for $^{12}C^{14}N^-$ and $^{12}C^{15}N^-$, respectively. Scale bars are 5 μm.

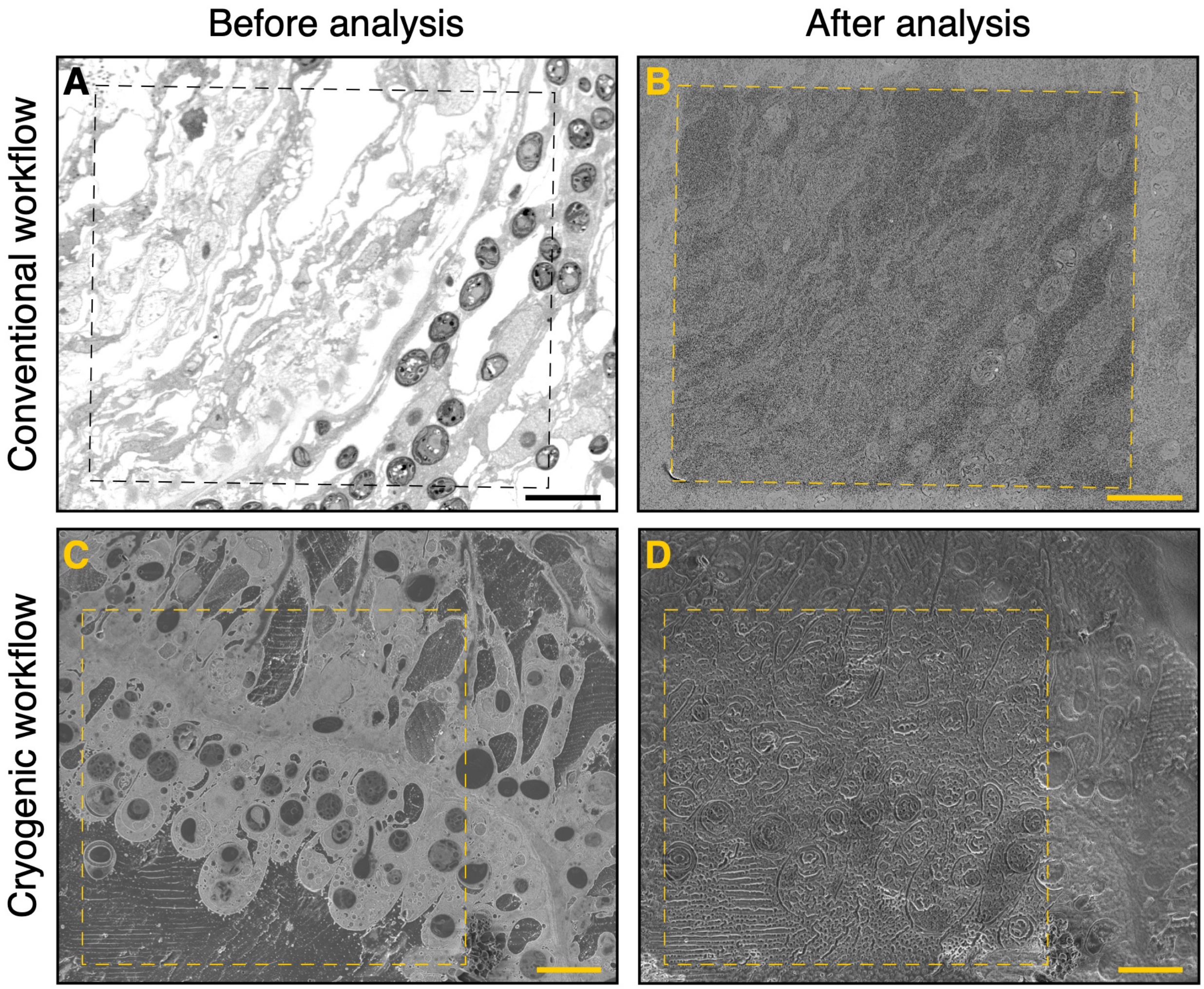

Figure S6. Sample surface before and after NanoSIMS imaging. SEM images of sample surfaces before and after NanoSIMS analysis with conventional (**A** and **B**) and cryogenic (**C** and **D**) workflows, respectively. Squares indicate the areas of NanoSIMS analysis. In A and C the sample surfaces were coated with about 3 nm metal, whereas in B and D the surface was coated with about 20 nm metal, except for the areas of NanoSIMS imaging, where this coating had been removed by pre-sputtering. Scale bars are 10 µm.
